# Alkanes as Membrane Regulators of the Response of Early Membranes to Extreme Temperatures

**DOI:** 10.3390/life12030445

**Published:** 2022-03-17

**Authors:** Loreto Misuraca, Antonino Caliò, Josephine G. LoRicco, Ingo Hoffmann, Roland Winter, Bruno Demé, Judith Peters, Philippe M. Oger

**Affiliations:** 1 University Grenoble Alpes, CNRS, LIPhy, 38000 Grenoble, France; misuraca.loreto@gmail.com; 2 Institut Laue Langevin, 38042 Grenoble, France; hoffmann@ill.fr (I.H.); deme@ill.fr (B.D.); 3 INSA Lyon, Université de Lyon, CNRS, UMR5240, 69100 Villeurbanne, France; antonino.calio@insa-lyon.fr (A.C.); josephineloricco@gmail.com (J.G.L.); 4 Fakultät für Chemie und Chemische Biologie, Physikalische Chemie, Technische Universität Dortmund, 44227 Dortmund, Germany; roland.winter@tu-dortmund.de; 5 Institut Universitaire de France, 75005 Paris, France

**Keywords:** protomembranes, origin of life, thermal stability, alkanes, neutron scattering, DLS, FTIR

## Abstract

One of the first steps in the origin of life was the formation of a membrane, a physical boundary that allowed the retention of molecules in concentrated solutions. The proto-membrane was likely formed by self-assembly of simple readily available amphiphiles, such as short-chain fatty acids and alcohols. In the commonly accepted scenario that life originated near hydrothermal systems, how these very simple membrane bilayers could be stable enough in time remains a debated issue. We used various complementary techniques such as dynamic light scattering, small angle neutron scattering, neutron spin-echo spectroscopy, and Fourier-transform infrared spectroscopy to explore the stability of a novel protomembrane system in which the insertion of alkanes in the midplane is proposed to shift membrane stability to higher temperatures, pH, and hydrostatic pressures. We show that, in absence of alkanes, protomembranes transition into lipid droplets when temperature increases; while in presence of alkanes, membranes persist for longer times in a concentration-dependent manner. Proto-membranes containing alkanes are stable at higher temperatures and for longer times, have a higher bending rigidity, and can revert more easily to their initial state upon temperature variations. Hence, the presence of membrane intercalating alkanes could explain how the first membranes could resist the harsh and changing environment of the hydrothermal systems. Furthermore, modulating the quantity of alkanes in the first membranes appears as a possible strategy to adapt the proto-membrane behavior according to temperature fluctuations, and it offers a first glimpse into the evolution of the first membranes.

## 1. Introduction

The origin of life on Earth occurred approximately between 4.4 (first evidence of liquid water and of an atmosphere [1]) and 3.5 (oldest dated microfossils [2]) billion years ago. The early Earth would be far from resembling our planet as we know it today, with an early atmosphere possibly being similar to that of current Venus [3] and to a much higher geothermal heat flow [4]. The Earth Archaean crust would have been comprised of a multitude of ubiquitously distributed hydrothermal systems [5,6] (vents [7] or fields [8]). Hydrothermal vents and fields are among the most accepted environments within which life could have originated, because the thermal and chemical gradients would have provided a source of energy long before the proto-living systems could have been able to harvest it themselves.

Both types of hydrothermal systems share high temperature as a common feature (T = 85–110 °C at the terrestrial surface [9], 50–350 °C in the proximity of the vents [10]). Deep-sea hydrothermal vents are also a system subject to high hydrostatic pressure (up to p = 1000 bar) and although hydrostatic pressure is known to produce effects opposing those of high temperature, (e.g., on multi-lamellar vesicle (MLV) structure and dynamics [11,12,13,14,15]), this would only partially counter-balance the temperatures reached at the vents. The high temperatures may actually have been key for specific reactions necessary for the emergence of life [16,17], in particular for the synthesis of membrane forming molecules such as single chain amphiphiles (SCAs) through Fischer–Tropsch Type (FTT) reactions [18,19]. It is worth noting that, along with SCAs such as short-chain fatty acids and alcohols, other products of FTT reactions are n-alkane and n-alkene species [18,19]. Alternatively, Bonfio et al. have shown recently that exposing glycerol-2-phosphate to acylating agents leads to the formation of a library of acylglycerol-phosphates through energy-dissipative cycling [20].

Due to the simple reaction pathways required to synthesize SCAs in a prebiotic environment (as opposed to more complex phospholipids [21]), vesicles made by short, single chain carboxylic acids and n-alkanols are the most extensively studied systems for protocell membranes [22,23]. Previous studies have shown that short-chain fatty acids are able to self-assemble into membrane compartments [24] when specific requirements of solution pH and concentration are met [25]. In particular, fatty acid vesicles form at concentrations above a critical vesicle concentration (CVC) and at pH close to the fatty acid dissociation constant (pKa), both quantities being functions of the fatty acid chain length [25]. Mixing the fatty acid with same chain fatty alcohol has the effect of significantly decreasing the CVC [25] and broadening the pH range of vesicle existence [13]. Furthermore, our previous study [26] demonstrated that while a pure fatty acid sample above the CVC always shows a coexistence between vesicles and micelles, a mixture of the same fatty acid with the corresponding alcohol gives only vesicle structures at ambient temperature, further demonstrating the strong stabilizing impact that alcohol has on the membrane. Moreover, mixtures of decanoic acid, decanol, and geraniol (an isoprenoid alcohol) were found to improve the vesicle resistance towards ionic solutes relevant to oceanic hydrothermal environments [27].

While the high temperature conditions close to the hydrothermal systems are seen as favorable to catalyzing the synthesis of the building blocks necessary for life (including SCAs), these same conditions also pose a critical concern in the definition of SCA vesicles as plausible protomembrane candidates. It is unclear how the self-assembled compartments (vesicles) would have been able to maintain their structure and functionality at high temperature. The increase in thermal energy has been shown to cause the dissolution of pure fatty acid vesicles into micelles above 60 °C [26]; in the case of the fatty acid–alcohol mixtures, it inhibits the membrane capability to retain oligonucleotides [16] and triggers a conformational transition in the vesicles [26]. Thus, it is still unclear how protomembranes could evolve in a context that was unfavorable to their intrinsic stability.

High temperature is also a challenging parameter for more complex, modern lipid membranes [28]. We can find suitable strategies to overcome the issue by observing the structure of the membrane in contemporary hyperthermophilic organisms. For instance, strategies in the archaeal domain for temperature adaptation include three main lipid modifications: the use of branched hydrophobic chains (polyisoprenoid vs. linear alkane); the ether bonding of the chains to the glycerol (instead of an ester bonding) [29]; the use of bipolar tetraether lipids which can form monolayers [30]. These modifications create a monolayer membrane, which is a more rigid, impermeable, and temperature-stable membrane, capable of sustaining life in the hydrothermal vent environment for these Archaea. More recently, it has been proposed that a small fraction of apolar polyisoprenoid hydrocarbons inserted into the midplane of the membrane of *Thermococcus barophilus* could explain the temperature stability of its membrane bilayer [31,32,33,34]. Most of the strategies put in place by the modern extremophiles require the use of complex organic molecules which were certainly not readily available in the prebiotic environment, with the notable exception of the small fractions of apolar (linear) alkanes, which can be synthesized by the FTT reactions which also generate the fatty acids and alcohols.

The possibility that apolar hydrocarbon molecules enter lipid bilayers—and by doing so modify the membrane characteristics and properties—has received attention in previous studies, some of them in close relation to the study of the origin of life. Deamer and coworkers [35,36] proved that polycyclic aromatic hydrocarbons can be inserted into membranes made of decanoic acid and hypothesized their role as pigment systems for a primitive form of photosynthesis. Such a possibility was further confirmed by Cape et al. [37]. Salvador-Castell and coworkers [32,33] found that squalane (C30 isoprenoid) can enter an archaea-like lipid membrane by positioning itself in the bilayer mid-plane and can induce non-lamellar phases as a function of temperature and hydrostatic pressure by modifying the membrane curvature. McIntosh et al. [38] showed that C6–C16 linear alkanes can be inserted into phospholipid DMPC (1,2-dimyristoylphosphatidylcholine) and DPPC (1,2-dipalmitoylphosphatidylcholine) bilayers and modify the membrane phase transition temperatures.

In a recent study, we explored the same type of samples as a function of temperature and high hydrostatic pressure and found that alkanes strongly impact the multilamellar vesicle ordering and chain dynamics within the membranes formed by a decanoic acid-decanol mixture and in presence or absence of linear (eicosane, C20) or isoprenoid (squalane, C30) alkanes [34]. Here, we are further characterizing the protomembrane properties and their stability at temperatures relevant to the hydrothermal systems, e.g., higher than 60 °C.

For that, we explore the temperature range T = 20–80 °C, which is more relevant to the conditions near the hydrothermal systems, with a special focus on the identification of the high temperature (T ≥ 60 °C) phase and properties and show that both linear and isoprenoid alkanes have clear effects in the thermal stability of the model protomembrane, shifting it to higher temperatures. Our results demonstrate that apolar hydrocarbons, in particular prebiotically available linear alkanes, could have constituted a suitable strategy for the protomembranes to survive the high temperature conditions imposed by the hydrothermal environments.

## 2. Materials and Methods

### 2.1. Sample Preparation

The samples were prepared following the protocol described in [26]. Sodium decanoate, 1-decanol, eicosane, squalane, bicine buffer, and D_2_O were purchased from Sigma Aldrich-Merck (Darmstadt, Germany). Mixtures of 1:1 sodium decanoate and decanol were dissolved in a chloroform:methanol 1:1 mixture, together with the appropriate amount of alkanes when used. After solubilization, samples were dried under a nitrogen stream and left overnight in a desiccator with vacuum pumping. Samples were checked gravimetrically at every step to ensure the complete evaporation of the chloroform:methanol mixture.

The buffers were prepared by dissolving 0.2 M of bicine salt in H_2_O (for DLS experiments) or D_2_O (for FTIR, SANS, NSE experiments) and titrated to pH 8.5 (pD 8.5 for the deuterated solution). All buffers were filtered through a 0.2 μm Millipore membrane before use.

The dried decanoic acid decanol (and eventually the alkane) films were suspended in buffer to obtain an 80 mM concentration for each sample and vortexed at maximum speed for ≈2 min, until all samples showed no aggregates and the suspensions appeared milky (characteristics of large vesicle formation).

Afterwards, the samples were extruded using the mini-extruder from Avanti Polar Lipids (Alabaster, AL, USA), using a 100 nm pore size polycarbonate membrane. The extrusions, undergoing 11 passes per sample, were performed with the heating block in thermal equilibrium at 40 °C and less than 24 h before every experiment.

### 2.2. Dynamic Light Scattering (DLS)

The DLS experiments were performed on a Zetasizer Nano ZS90 (Malvern Panalytical Ltd., Malvern, UK) available at the Institut Laue-Langevin (ILL) Grenoble (France), with an incoming laser light of λ = 633 nm (He-Ne). The samples, extruded to minimize polydispersity and multilamellarity, were loaded into 200 μL quartz cells. The data, acquired at a 90° fixed angle as a function of temperature, were collected continuously and then grouped by 3 runs of 30 s to allow for error estimations.

The resulting DLS autocorrelation functions g_2_(t) were analyzed following a method developed and described in [39] in order to extract the Brownian diffusion coefficients D. The model function (Equation (1)) has the form

g_2_(t) − 1= B + β exp(−2Dq^2^t)(1−μ_2_/2)^2^ (1)

with Dq^2^ = Γ (decay rate) the first moment, μ_2_ (related to the polydispersity) the second moment, B and β background and scaling factor, respectively.

Then, from each diffusion coefficient D, one can estimate the corresponding hydrodynamic radius R_h_ using the Stokes–Einstein equation (Equation (2))

D = k_B_T/(6πR_h_η) (2)

where k_B_ is the Boltzmann constant, T the temperature, η the solvent viscosity (assumed as the one of H_2_O).

### 2.3. Small-Angle Neutron Scattering (SANS)

SANS experiments were carried out at the ILL (Grenoble, France) using the D33 instrument [40]. Three configurations combining incident wavelengths λ (5 Å and 14 Å) and detector distances (2, 10, and 12 m) were used, corresponding to a range of momentum transfers 0.001 Å^−1^ < q < 0.5 Å^−1^.

The measurements were performed using a thermostated sample changer allowing a temperature range of 20 to 80 °C. The scans proceeded in the following order: (1) C10mix; (2) C10mix + 2% h-eicosane; (3) C10mix + 2% h-squalane. The samples were kept for a total of ≈2 h at each temperature point.

For each SANS curve, analyzed using the SASView software 4.2.2 (http://www.sasview.org/), different model form factors (and when necessary, a combination of them) were employed, depending on the need. The full formulas for the theoretical form factors, all available from SASView, can be found in the Supplementary Materials or in [41,42]. In particular, the normalization of the SANS data to absolute scale allowed us to quantify and follow the fraction of lipid self-assembled into bilayers at every measured temperature and for every sample, as well as the fraction of sample aggregating into lipid droplets.

### 2.4. Neutron Spin Echo (NSE) Spectroscopy

NSE spectroscopy measurements were performed on the instrument IN15 [43] at ILL. The samples, extruded and placed into 1 mm thick Hellma cells, were measured in the 5° angle configuration, leading to an observable q range of 0.04 Å^−1^ < q < 0.07 Å^−1^. Each sample was measured at the temperatures T = 20, 50, 60, 70 °C. An in situ DLS available on IN15 allowed tracking of the light scattering intensity traces and the vesicle diffusion coefficient of all samples during the measurements. The samples were placed into an automatic sample changer regulated with a thermal bath; therefore, they were brought to and kept at each temperature point for the same amount of time (≈4 h per temperature). The measurements followed the order: (1) C10mix; (2) C10mix + 2% h-eicosane; (3) C10mix + 2% h-squalane.

The data obtained from NSE were analyzed in the framework of the Zilman–Granek theory [44] to model the membrane fluctuations. Each intermediate scattering curve F(q, t) was fit with a function (Equation (3)) of the form

F(q, t) = exp(−Dq^2^t) × exp(−(Γ_ZG_q^3^t)^2/3^) (3)

The first term models the entire vesicles’ Brownian diffusion with diffusion coefficient D. This parameter is obtained from the in situ DLS data and it is then used as a fixed parameter during the NSE fits. The second term corresponds to the membrane relaxation: it is modeled as a stretched exponential with decay rate Γ_ZG_. The latter has the expression [44] of Equation (4)

(4) $$\Gamma_{\mathrm{ZG}}=0.025\gamma \sqrt{\frac{k_{B}T}{\overset{ˇ}{\kappa }}}\frac{k_{B}T}{\eta },$$

with $\overset{ˇ}{\kappa }$ being the effective bending/compression modulus, $k_{B}$ the Boltzmann constant, η the D_2_O viscosity, γ ≈ 1 for $\overset{ˇ}{\kappa }\gg k_{B}T$. From $\overset{ˇ}{\kappa }$, one can find the actual membrane bending rigidity estimates, using a correction from Seifert and Langer [45] to account for density fluctuations in the membrane [46] (Equation (5))

(5) $$\overset{ˇ}{\kappa }=\kappa \left(1+24\frac{2d_{\mathrm{mono}}^{2}}{d_{\mathrm{bilayer}}^{2}}\right),$$

with κ being the bending rigidity, $d_{\mathrm{bilayer}}$ the bilayer thickness, and d_mono_ the height of the monolayer neutral surface [47]. The quantity d_mono_ is generally unknown, although it ranges in d_bilayer_/4 ≤ d_mono_ ≤ d_bilayer_/2. This quantity has the practical consequence of rescaling $\overset{ˇ}{\kappa }$, to obtain the bending rigidity constant κ. In the following, we will consider for d_mono_ the hydrocarbon chain length found from previous NMR experiments on the C10mix membrane (L_chain_ ≅ 8.3 Å [26]). This is equivalent to modifying Equation (4) by substituting $\overset{ˇ}{\kappa }$ →κ and the prefactor 0.025→0.0082, a value that is similar to the most accepted value for phospholipid-based vesicles (0.0069 [48]) to get Equation (6)

(6) $$\Gamma_{\mathrm{ZG}}=0.0082\gamma \sqrt{\frac{k_{B}T}{\kappa }}\frac{k_{B}T}{\eta }.$$

To analyze the NSE data, some considerations can be made based on knowledge of the instrument time (/energy) window. From Equation (3), one can see that the term related to the Brownian diffusion, namely exp(−Dq^2^τ), tends to a constant for small enough values of the diffusion coefficient D: exp(−Dq^2^τ) ≈ 1 when D ≈ 0. Therefore, we will neglect the contribution of the Brownian diffusion coming from the largest assemblies as detected from the in situ DLS, and consider only the structures that give the lowest R_h_ at temperatures where the coexistence is observed, T = 60 and 70 °C. In practice, this means that the diffusion of the large lipid droplets has negligible effect on the NSE data analysis, while the diffusion of the vesicles is considered and disentangled from the membrane relaxation signal. This strategy is also supported by what we observed by SANS, where the region q > 0.04 Å^−1^ is clearly dominated by the membrane contribution (as a reminder, the NSE measurements were performed in the range 0.04 Å^−1^ < q < 0.07 Å^−1^).

Concerning the possible contribution of the large lipid droplets in the NSE F(q, t), one can assume it as an elastic constant, which would lead to a modification of Equation (3) as follows (Equation (7)):

F(q, t) = I_vesicles_ × exp(−Dq^2^t) × exp(−(Γ_ZG_q^3^t)^2/3^) + I_droplets_ (7)

with I_x_ being the intensity ratio of population x in the NSE q-range. However, by plotting the curves in a log[F(q, t)/exp(−Dq^2^t)] vs. (q^3^t) ^2/3^ representation, we can show (Figure 5) that all data at T = 70 °C fall extremely well onto a linear master curve (having a slope m = −Γ_ZG_^2/3^). This ensured that all contributions from the lipid droplets could be neglected in the analysis and that the model function of Equation (3) was sufficient to fit the data.

### 2.5. Fourier Transform Infrared (FTIR) Spectroscopy

The FTIR spectra were acquired on a Vertex70 FTIR spectrometer (Bruker, Berlin, Germany) with 1 cm^−1^ resolution. The temperature steps, as well as the time at which the samples were kept at each temperature, were set to match the NSE experiment on IN15 (≈4 h per temperature). Samples were followed by acquiring FTIR spectra every 15 min. Every spectrum was automatically averaged among 256 collections by the instrument software.

The data were analyzed following a specific frequency peak at ν_symm_ ≈ 2850 cm^−1^. This peak, corresponding to the CH_2_ symmetric stretching frequency, was chosen for its well resolved position in the FTIR wavelength range. The position of the peak was evaluated by fit with one (or two, when coexistence was observed) Gaussian functions in the vicinity of the peak. The temperature and time dependence of the peak provides information about the lipid tail’s conformation and vibrational dynamics.

## 3. Results

### 3.1. Effect of Temperature on Conformational Changes

**Figure 1:**
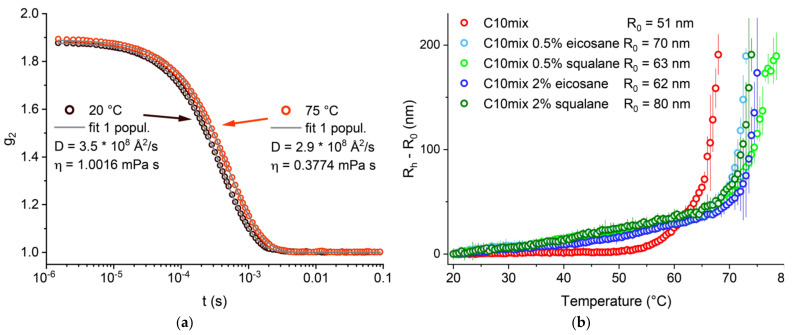
(**a**) Example of autocorrelation functions, for the sample C10mix at T = 20 and 75 °C respectively, with the corresponding diffusion coefficients D found from the fits. Viscosity data were adapted from [49]. (**b**) R_h_ − R_0_ (R_0_: radius at T = 20 °C) as a function of T for all measured samples. C10mix data were adapted from [26]. Values are displayed up to R_h_ − R_0_ ≈ 200 nm, above which the polydispersity was likely too high to give reliable quantitative estimations.

We first studied and compared our three model systems (C10mix, C10mix + eicosane, and C10mix + squalane) by following the dependence of the vesicle mean size with temperature. This was done by performing DLS measurements to exploit the vesicle Brownian diffusion to probe their average size. The samples were extruded with a 100 nm pore membrane, to start with well monodispersed suspensions. An example of the autocorrelation function for the C10mix at two different temperatures is given in Figure 1a. The vesicle hydrodynamic radius (R_h_) was determined from the diffusion coefficient D extracted from the fits of the DLS autocorrelation functions as described in Section 2. The change in R_h_ is shown as a function of temperature. At elevated temperatures, there is a steep increase in the R_h_ with temperature corresponding to a conformational transition to larger macrostructures which was observed and described previously [26]. A clear difference between the C10mix sample lacking any alkane, as compared to all the others, is observed. In particular, the alkanes are found to shift the conformational transition of the C10mix to a higher temperature (55–60 °C for the C10mix, 65–70 °C for the others). Furthermore, the alkanes modify the rate of vesicle size increase at low temperature (20 °C < T < 50 °C), leading to a steeper R_h_ increase in that range. Interestingly, the retardation effect of linear (eicosane) and branched (squalane) alkanes at either 0.5% or 2% are the same within the precision of the technique employed. These results give a clear first indication that both types of alkane increase the vesicle thermal stability.

### 3.2. Amphiphile Partitioning

To analyze the nature of the macro-structures formed by the SCAs at every temperature in more detail, SANS experiments were performed. SANS allows discrimination between different types of macrostructures thanks to its wide q-range. The quantity q, known as the scattering vector, can be thought as the equivalent in the reciprocal (Fourier) space of the distance variable (d) in the real space. Thus, a wide q-range translates into a wide range of length-scales, which can be probed with SANS by analyzing the q dependence of the scattering intensity. This allows us to distinguish, for instance, between dense spheres, cylinders, hollow spheres, etc. Moreover, quantitative information about the average sizes, polydispersity, shell thickness of the hollow spheres and membrane correlations (if present) can also be obtained.

**Figure 2:**
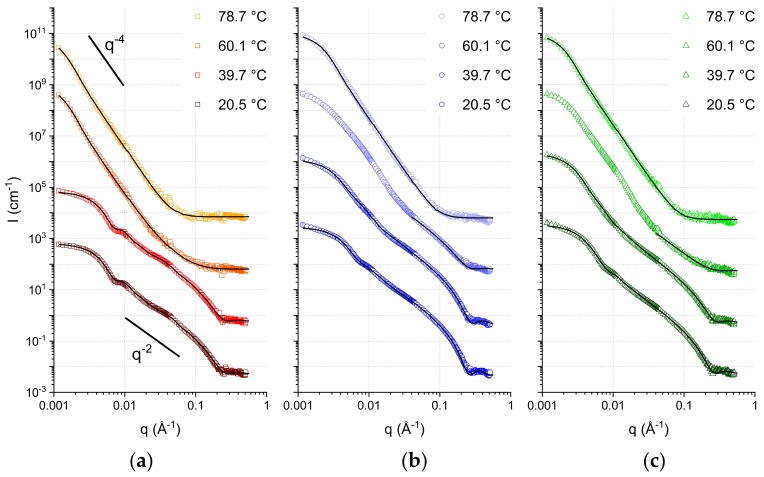
SANS curves of extruded samples at the temperatures investigated. (**a**) C10mix sample. (**b**) C10mix + 2% h-eicosane sample. (**c**) C10mix + 2% h-squalane sample. The black lines are the best fits to the data. The curves are vertically shifted for clarity.

In Figure 2, the collected SANS curves are shown together with the best fits obtained. From a first inspection of the curves, it is possible to see some distinct features:

- The curves at lower temperatures reach a plateau value in the lowest q-range. This denotes the so-called Guinier regime [41]. Its occurrence indicates the presence of a monodisperse population of spherical objects, a result of the extrusion process. The low-q data can therefore be used to calculate the average object size. In our case, these values are calculated directly by fitting the curves with models contained in the software SASview that cover the entire q range explored.
- Mostly at the lowest temperature, but also partially at the higher ones, the region 0.05 < q < 0.15 Å^−1^ follows a q^−2^ decay. This trend is compatible with lamellar form factors (vesicles), where the thickness of the membrane is measurable through the position of the minimum occurring at slightly higher q values (in our data, visible at 0.2 < q < 0.3 Å^−1^; the resulting membrane thickness values can be found in the Supplementary Materials).
- The high temperature data clearly show the predominance of a significantly steeper decay, with a q^−4^ power law. This decay, also known as Porod law [50], originates from well-defined, smooth interfaces, and is the signature of large aggregates modeled here with a spherical shape. Some of the intermediate temperatures exhibit a coexistence of q^−4^ decay in the lowest q region together with a q^−2^ decay in the intermediate-higher q region.

**Figure 3:**
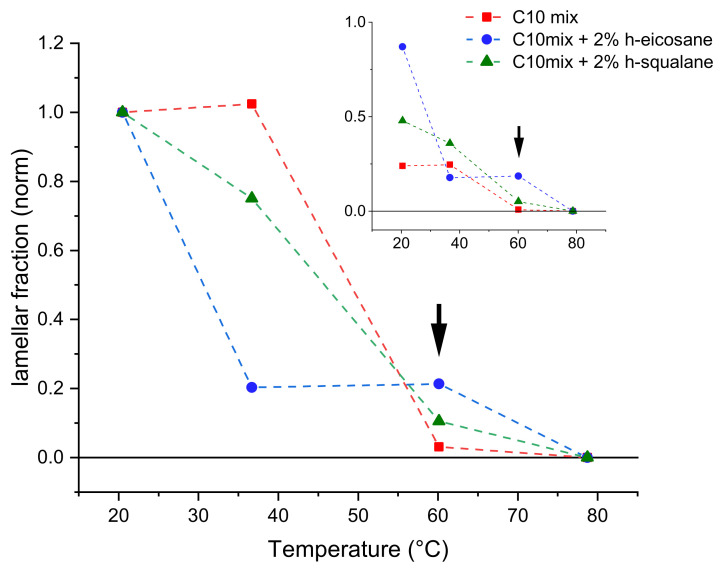
Fraction of the lamellar form factors obtained from fits to every sample and temperature point studied, normalized to the initial value at T = 20 °C. The inset shows the data normalized to the sample volume fraction. Arrows point to the T = 60 °C data, where a significantly higher fraction of lamellar phase is observed on the alkane containing samples.

The coexistence of two distinct assemblies (bilayers and droplets, respectively) can be judged qualitatively (by visual inspection of the curves) as well as quantitatively (by fitting the SANS data). In particular, because of the faster decay of the droplet signal (q^−4^), the lamellar form factor is dominating in the region at q > 0.04 Å^−1^: this allows for a straightforward discrimination of the fraction of sample that self-assembles into bilayer structures, by proper scaling the q^−2^ in the middle-high q region (Table 1). Figure 3 shows the fraction of lipid forming bilayers as a function of temperature increase.

The most important difference is observed in the data acquired at T = 60 °C. At this temperature, the C10mix sample shows a very small fraction of lamellar assemblies, while most of the sample appears to have formed large dense spheres (droplets) because of the temperature increase. Instead, samples containing intercalated alkanes retain a larger fraction of lamellar structures at this temperature. Note that, at this temperature, the SANS curves of the samples containing eicosane and squalane (Figure 2) are found in an intermediate state where it was not possible to fit the fraction of droplets at low q with a single dense sphere form factor. For this reason, the fitting at T = 60 °C for these two samples was performed only in the q range where the lamellar form factor predominates (q > 0.04 Å^−1^), to estimate the corresponding fraction.

As Figure 3 shows, the loss of the lamellar phase fraction in favor of dense spheres from its initial value at T = 20 °C is more pronounced in samples containing the alkanes at T = 40 °C, while the situation is reversed at the higher temperature T = 60 °C. Finally, all samples have completed the transition towards the dense sphere assemblies at the highest measured temperature of T = 80 °C.

These results demonstrate the membrane stabilizing role of both kind of alkanes, up to at least T = 60 °C. Moreover, they show that the result of the high temperature conformational transition on ULV systems is the formation of a lipid droplet phase.

### 3.3. Membrane Bending Rigidity

As the above experiments have demonstrated, the presence of alkanes in the membrane has a significant impact in modifying the phase boundaries and structural properties of the protomembranes. To gain further insight into these effects, we looked at the consequences of temperature on membrane dynamics using NSE spectroscopy performed on extruded vesicles at temperatures in the range 20 °C < T < 70 °C.

NSE spectroscopy is a technique that allows the studies of dynamic modes with very long timescales (up to 1 ms), for example the collective motions that lead to membrane undulations [44,45,46]. These fluctuations are a function of the bending rigidity of the membranes, and therefore can be followed by NSE using the formalism described in Section 2.

Prior analysis was performed on the data acquired by in situ DLS during the NSE measurements. As opposed to the DLS experiments performed offline (data in Figure 1), in this case the autocorrelation functions are averaged over the duration of the NSE scan (≈4 h per T step); therefore, generally different results and some cases of coexistence between different size populations are to be expected.

**Figure 4:**
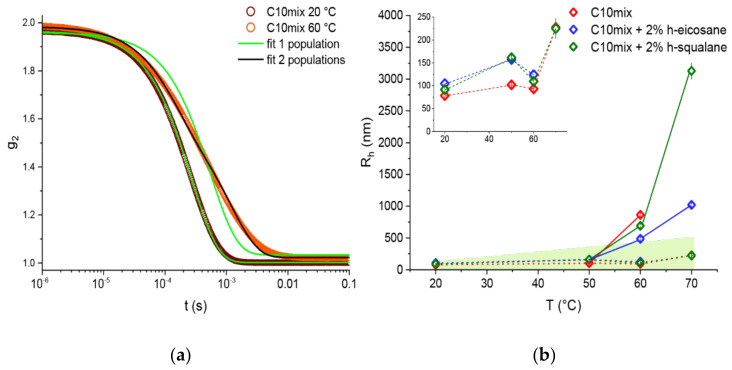
(**a**) Example of autocorrelation curves collected for the C10mix sample using the in situ DLS at T = 20 and 60 °C, respectively. Green lines show fits using a simple exponential decay function; the black curve is a fit of the T = 60 °C data with a sum of two exponential functions. (**b**) Resulting R_h_ values for the two populations, one represented by a dashed line, the other by full lines; outside the green region, the factor exp(−Dq^2^τ) ≥ 0.95 (D ≲ 10^−1^ Å^2^/ns) and the diffusive contributions will be neglected. Note that the limit in R_h_ changes with T because it scales with the solvent viscosity. Inset: vertical zoom for R_h_ ≤ 250 nm.

Figure 4 shows examples of autocorrelation functions at different temperatures as well as the R_h_ calculated for each population, temperature, and sample probed. Two populations are needed to fit the data at the two highest temperatures measured, T = 60 and 70 °C, with the only exception of the C10mix + 2% eicosane at T = 70 °C. These results are in line with what we found by SANS, namely that the SCAs tend to self-assemble into both vesicle and dense sphere structures when reaching high temperatures. Additionally, from the SANS data (Table 1), one can see that the droplets have a much larger average size with respect to the initial size constrained by the extrusion. Therefore, it is likely that the smaller R_h_ values observed using the in situ DLS (Figure 4) are related to the vesicle assemblies, while the higher R_h_ values correspond to the lipid droplets instead.

**Figure 5:**
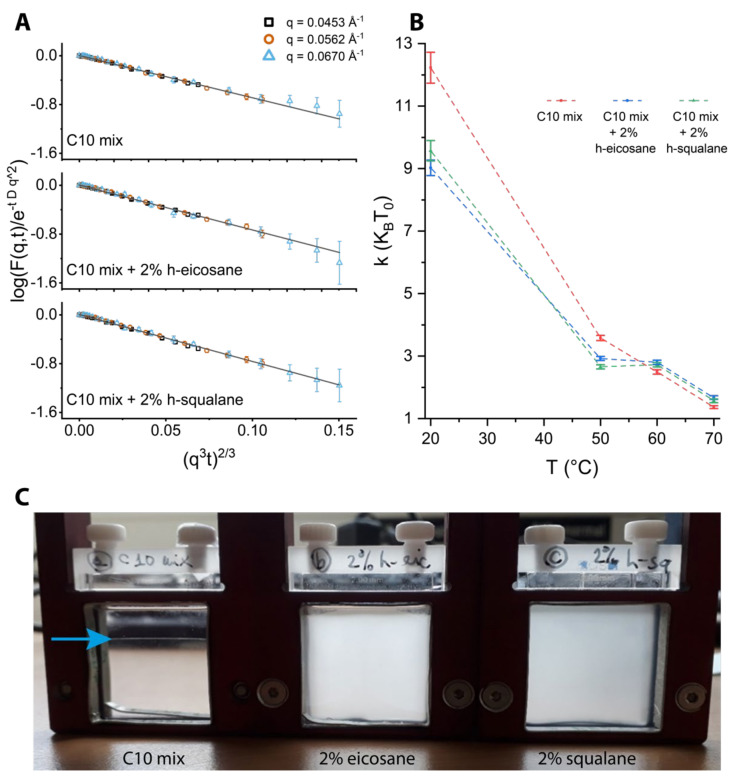
(**A**) NSE data at T = 70 °C plotted in the representation log[F(q, t)/exp(−Dq^2^t)] vs. (q^3^t) ^2/3^. This highlights the stretched exponential decay predicted by the Zilman–Granek theory [44] and additionally shows that a more complex model taking into account the lipid droplet signal is unnecessary. (**B**) Plot of the bending rigidity estimates as a function of the temperature. (**C**) The sample appearance after the NSE thermal scans. The blue arrow points to the interface between the water and lipid phases.

Due to the size distribution of the droplets and from the theoretical definition of the intermediate scattering function (shown in Figure 5a), NSE data give us selective information on the membrane phase and are not affected by the presence of the lipid droplets. The results in Figure 5b allow for several interpretations. First, there is a clear temperature effect on lowering the membrane rigidity, as expected. In line with the aforementioned experiments, the data show marked differences between the samples lacking or containing alkanes in the membrane. In particular, the rigidity is lowered by the addition of the alkanes at lower temperatures, while the trend is inverted at 60 and 70 °C with a small alkane-mediated membrane stiffening. Eicosane and squalane give results comparable to one another. The difference at high temperature between the C10mix and the samples with the alkanes is significant in terms of the data and the associated errors. However, (assuming a correct scaling of the bending rigidity, see Section 2) this difference is a small fraction of 1 k_B_T, questioning the actual physical significance of such difference.

A striking difference between the samples with or without alkanes was found by visual inspection of the suspensions after the temperature ramp (Figure 5c). The samples are indistinguishable from one another when prepared, but at the end of the scan the C10mix had lost its turbid appearance, and instead an interface was visible denoting a macroscopic phase separation. In contrast, the samples with alkanes were single phased and maintained turbidity, which is typical of samples containing vesicles.

The results show that the alkanes significantly affect the protomembrane bending rigidity: at low temperature, the rigidity is lowered by the alkanes, while the difference is negated (and instead the rigidity is slightly higher) at high temperatures.

### 3.4. Time Evolution of Chain Conformation and Dynamics

As discussed, the NSE data show a significant impact of the alkane molecules in modifying the collective dynamics of the amphiphiles constituting the model membranes, encoded in the effective bending rigidity parameter. It is now useful to investigate whether this impact is also visible at a much smaller length scale, i.e., in the vibrational dynamics of each SCA induced by thermal energy. For that, FTIR spectra were acquired on samples with 1% perdeuterated alkanes to focus on a specific vibrational mode of the decanoic acid and decanol acyl chains (in our case, the symmetric stretching of CH_2_ groups was chosen) at the same temperature points that were considered in the NSE experiment.

The wavenumber of this band is conformation-sensitive and thus responds, for example, to temperature-induced changes of the trans/gauche ratio in acyl chains. In fact, the position of the symmetric CH_2_ stretching vibration is a measure of the number of gauche conformers in the acyl chains. When all methylene groups are in the trans conformation (e.g., in the ordered gel phase of the di-C16 DPPC bilayer), the band is observed around 2849 cm^−1^. Addition of gauche conformers, accompanied by increased vibrational dynamics, results in a shift to higher frequency. At the main transition from the gel to the fluid-like (liquid-crystalline) phase, which results in melting of the acyl chains, the band shifts by 2–3 cm^−1^, reaching about 2852 cm^−1^ in the all-fluid phase of the pure lipid bilayer DPPC [51].

**Figure 6:**
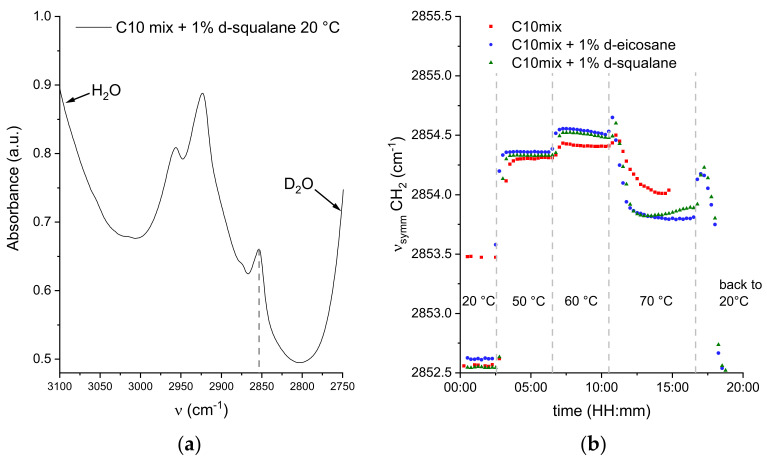
(**a**) Example of a FTIR spectrum for the C10mix + 5% h-squalane at T = 20 °C, in the range of interest where the symmetric and asymmetric stretching vibrations of CH_2_ and CH_3_ are found. The dashed line indicates the position of the CH_2_ symmetric stretching mode at ν_symm_ ≈ 2852 cm^−1^. (**b**) ν_symm_ as a function of the time, following various temperature-jumps for all the samples.

Profiting from the much higher speed of acquisition with FTIR spectroscopy, the spectra were acquired continuously every 15 min, with jumps in temperature following the same timescale as in the NSE experiments. Figure 6 shows the position of the CH_2_ symmetric stretching peak as a function of experimental time for selected steps in temperature.

The first indication of the data is that the symmetric stretching frequency ν_symm_ quickly stabilizes to a specific value for all temperatures, even at the highest value of 70 °C. This confirms the equilibrium values found by NSE, regardless of the longer acquisition time per temperature point. Most of the spectra at 20 °C are best fit by a sum of two contributions, one at ν_symm_ ≃ 2852.5 cm^−1^ and the other at ν_symm_ ≃ 2861 cm^−1^ (see Supplementary Materials). In a few cases for the C10mix sample, a single contribution at ν_symm_ ≃ 2853.5 cm^−1^ was sufficient to describe the data. A coexistence of peaks is likely to be a sign of phase coexistence, such as a gel-to-fluid phase transition region, known to occur in the C10mix at T ≃ 10 °C [13,26].

The vibrational dynamics of the pure C10mix appears to be slightly lower than in the other samples at T = 50 and 60 °C, while an inversion occurs at 70 °C. Generally, an increase in ν_symm_ indicates enhanced conformational dynamics and lipid chain disorder. Interestingly, a marked decrease in the CH_2_ symmetric stretching frequency is observed at 70 °C, which appears to constitute a conformational and dynamic signature of the structural transition identified by the scattering data discussed above (Figure 1 and [26]).

The fast cooling of the alkane containing samples back to 20 °C shows the dynamic reversibility of the latter transition: a first increase towards the value characteristic of 50–60 °C, followed by a decrease until the same initial value of ν ≃ 2852.5 cm^−1^ at 20 °C is reached. The peak coexistence at 20 °C is also fully recovered (Supplementary Materials).

The data show that the membranes that incorporate the alkanes have a slightly higher lipid chain disorder at T ≤ 60 °C. The dynamics observed at T = 70 °C, dominated by the lipid droplet phase, show that the alkanes may increase the lipid packing inside the droplets.

## 4. Discussion

Our data show that the alkane incorporation significantly impacts the structure, topology, and dynamics of model protomembrane vesicles composed of the C10 acid and alcohol in response to temperature variations, in the range 20 °C < T < 80 °C. The linear (eicosane) and branched (squalane) alkanes studied in this work gave very similar results in all the experiments performed.

The main effects of the alkanes on vesicle behavior can be summarized as follows:

- Vesicle size vs. T: incorporation of alkanes leads to a larger size increase at low T, up to T ≈ 55–60 °C. Beyond that temperature range, a morphological and dynamical transition occurs for the C10mix [26], which is shifted to higher temperatures when the alkanes are added (T ≈ 65–70 °C).
- Amphiphile partitioning: high temperature causes a splitting in the lipid self-assembly into two kinds of macrostructures: lamellar (bilayer vesicles) and dense spheres (lipid droplets). Alkanes affect the sample partitioning by allowing more of the lamellar fraction to persist at T = 60 °C.
- Membrane bending rigidity: higher bending rigidity is observed in the C10mix membrane at low temperatures (T = 20 and 50 °C); whereas the samples with eicosane and squalane have slightly higher rigidities at higher temperatures (T = 60 and 70 °C).
- Vibrational dynamics: alkanes maintain slightly higher CH_2_ dynamics at T ≤ 60 °C, indicated by a small increase in v_symm_, while the symmetric stretching frequency is significantly lowered at T = 70 °C, which must be due to structural/morphological transition to a more densely packed state with less conformational and dynamic disorder. The transition back to T = 20 °C is fully reversible for the samples containing alkanes.

The samples were proven to be dynamically stable at all temperatures studied for several hours, thus excluding any possible distortion in the data due to non-equilibrium behavior. All the observed effects imply that the inclusion of alkanes, even of the simple and prebiotically relevant type (e.g., eicosane), may constitute an effective physical advantage for simple prebiotic model membrane structures to sustain high and fluctuating temperatures. By shifting the temperature of the C10mix conformational phase transition (Figure 1), the alkanes stabilize the vesicles over a wider temperature range, thus mitigating vesicle fusion and phase separation with coexisting lipid droplets.

Surprisingly, the alkanes seem to have overall opposite effects in the lower temperature range: faster radius increase (Figure 1), lower membrane rigidity (Figure 5), in some cases slightly higher CH_2_ dynamics (at T = 50 and 60 °C, Figure 6). The reason for this might have to do with the fluidity of the alkane molecules themselves which depends on the temperature: in fact, the eicosane has a bulk melting point at 37 °C [52], while the squalane (liquid at ambient temperature) has a viscosity that lowers quickly with the temperature [53] (Supplementary Materials). Therefore, the stabilizing influence of the alkanes on the C10mix may require these molecules to be in a low-viscosity/fluid state, allowing more efficient mixing (increase in mixing entropy) with the components of the C10mix that can be provided by high temperatures.

In the study of membrane stability at high temperatures, an essential underlying variable is the duration for which every thermal stress is applied. This is clearly shown, for instance, by the need for two populations of scatterers to fit the DLS autocorrelation function when the counting time lasted for several hours (as in the NSE experiments, Figure 4). Conversely, a continuous increase in temperature with fast DLS measurements required only a single population even at the highest measured temperatures (Figure 1). This factor needs to be considered, particularly in cases where the used experimental techniques required long counting times, namely the SANS and NSE measurements. Nevertheless, the validity of all effects we observed at high temperatures is justified by the order at which the samples were measured, as specified in Section 2. The C10mix sample was the one that equilibrated for the shortest time before measurement at each temperature, and yet it showed significant variations compared to the other alkane-enriched samples. This ensures that the C10mix vesicles are indeed less thermally stable than the alkane containing counterparts are, and that the observed effects are not a consequence of equilibration time differences between the samples.

The striking difference in the appearance of the samples after the temperature scan in the NSE experiments (Figure 5c) indicates that the transition from the lipid droplet state back to the vesicle state at room temperature occurs quickly for the samples with alkanes which indeed seem to stabilize the phase, while the C10mix sample might have phase separated irreversibly in absence of re-mixing or it occurs at a much slower rate. The reversibility between the vesicle and droplet states has been receiving increasing attention lately, because it could help to exploit both the selective permeability properties of the membranes (vesicles) and the spontaneous sequestering capability of the droplets [54].

The phase change towards lipid droplets can also explain the unexpected decrease in the CH_2_ symmetric stretching frequency observed at T = 70 °C in the FTIR data (Figure 6). As opposed to NSE, the FTIR data do not disentangle the signal coming from the membrane forming SCAs with the ones condensated into lipid droplets. The amphiphiles within the droplets are probably slightly more densely packed than in the vesicular structures at 50 and 60 °C, leading to a decrease in the number of gauche conformers and a minor decrease in chain disorder, with a concomitant decrease in v_symm_, but not reaching v_symm_-values typical for the gel-like ordered phases seen at low temperatures. Upon addition of eicosane and squalane, this effect is more pronounced, which might be due to a more efficient lipid packing of the droplets of this multicomponent mixture.

We point out that our findings on the temperature-mediated coexistence between fatty acid vesicles and droplets is in very good agreement with the results of a recently published study [55]. Although using different systems and buffering agents, the authors of that study show the occurrence of the vesicle-droplet (and vice versa) transition using hot-stage epifluorescence microscopy. This offers a useful real-space image observation of the phenomenon in the 1–10 µm range that complements our reciprocal-space scattering data in the 1–100 nm range.

Our results imply that the inclusion of alkane molecules inside model protocell membranes tunes both structural and dynamical properties (physical and chemical) of the vesicles. In particular, the linear and branched alkanes (eicosane and squalane) have the effect of stabilizing the membranes, rendering them more temperature stable, up to at least T = 60 °C and for several hours.

The conformational change at T ≈ 55–60 °C resulting from vesicle fusion of the C10mix is shifted to T ≈ 65–70 °C when the alkanes are added. The lamellar (membrane) phase is protected by the alkane presence which allows this phase to remain present at T = 60 °C while the control sample phase separates into lipid droplets. The bending rigidity of the protomembranes is significantly modified by the alkane incorporation: at lower temperatures, the alkane high viscosity interferes with the packing of the SCAs in the bilayer, leading to softer membranes. At higher temperatures (T ≥ 60 °C) the SCAs and the alkanes are more efficiently mixed in the hydrocarbon region of the bilayer, canceling the observed difference at low temperature, and even making the alkane-containing membranes slightly stiffer. The data showing the mean CH_2_ vibrational dynamics also support this idea, pointing to an increase in chain disorder of the samples with alkanes for T ≤ 60 °C.

Therefore, our study suggests that different kinds of alkanes, including types readily available in an early Earth environment, can be incorporated into protomembranes made of short SCAs, not only complex hydrocarbons such as polycyclic aromatic hydrocarbons [35,36,37] or long-chain isoprenoids [31,32]. At low temperatures (T ≤ 50–60 °C), the membranes containing alkanes have higher lipid chain disorder and consequently a lower bending rigidity, and perhaps this is also what drives the faster increase in size seen by DLS (Figure 1). Conversely, at higher temperatures (T ≥ 50–60 °C) the alkanes are better mixed with the membrane chains, which, in turn, achieve similar (slightly higher) bending rigidity as the alkane-lacking membranes. The alkane incorporation stabilizes the bilayer at high temperatures so that the lipid lamellar phase is partially maintained up to at least T = 60 °C.

The inclusion might have served as a successful strategy (through combinatorial chemistry selection) which could have allowed the protomembranes to cope with harsh thermal conditions surrounding hydrothermal systems.

## 5. Conclusions

Our results demonstrate that apolar hydrocarbons—in particular, prebiotically available linear alkanes—can be readily inserted into a membrane composed of a mix of the C10 acid and alcohol. The effect of the alkanes is to maintain the lamellar structuring of the membrane at a higher temperature before phase separation of the SCAs into lipid droplets occurs. A significant difference in the membrane bending rigidity was found at the temperatures investigated, suggesting the possibility that the alkanes can be used to tune the physical properties of the protomembrane. Finally, the alkanes were found to increase the membrane chain disorder for T ≤ 60 °C. The inclusion might have constituted a suitable strategy for the protomembranes to survive the high temperature conditions imposed by the hydrothermal environments, and through combinatorial chemistry selection they could have allowed the protomembranes to cope with harsh thermal conditions surrounding hydrothermal systems. The possibility that such systems (SCAs–alkanes) can acquire a higher thermal stability has significant consequences in our understanding of the possible mechanisms that could eventually have produced a stable living proto-organism. In this view, the adaptation strategy of the modern hyperthermophile *Thermococcus barophilus* to high temperature—i.e., the quantity modulation of apolar lipids in the membrane—could be another evolutionary remnant of the first successful living forms.

## Figures and Tables

**Table 1:** Main parameters for the fits of the curves at T = 20 and 60 °C. The values for the fractions (lamellar and sphere) are normalized to the lipid volume fraction. ULV: Unilamellar Vesicle; BLV: Bilamellar Vesicle. * Since the Guinier regime is not fully reached at low q for the dense sphere form factors, the radius values found from these fits are not reliable and should be considered as minimum values instead. The full list of fitting parameters can be found in the Supplementary Materials.

*Sample*	C10mix		C10mix + 2% h-Eicosane		C10mix + 2% h-Squalane	
**Temperature (°C)**	**20.5**	**60.1**	**20.5**	**60.1**	**20.5**	**60.1**
Model	lamellar (ULV + BLV)	sphere + lamellar	lamellar (ULV + BLV)	lamellar	sphere + lamellar (ULV)	lamellar
q range (Å^−1^)	all	all	all	0.04–0.57	all	0.04–0.57
Lamellar fraction (×10^−3^)	239.0 ± 0.8	7.4 ± 0.2	869 ± 1	186.0 ± 0.6	478.3 ± 0.6	50 ± 2
Vesicle radius (Å)	412 ± 2 (ULV) 154 ± 6 (BLV)	-	389 ± 3 (ULV) 157 ± 3 (BLV)	-	397 ± 3	-
Sphere fraction (×10^−3^)	-	85.0 ± 0.2	-	-	458.3 ± 0.2	-
Sphere radius (Å)	-	768 *	-	-	516 *	-

## Data Availability

The raw neutron data is available upon request: D33 (DOI: 10.5291/ILL-DATA.9-13-905) and IN15 (DOI: 10.5291/ILL-DATA.INTER-464).

## Supplementary Materials

The following are available online at https://www.mdpi.com/article/10.3390/life12030445/s1: Additional information on SANS data analysis including Table S1 with a full list of SANS fit parameters. Information on the in situ DLS measurements taken on the NSE spectrometer including Figure S1 with in situ DLS intensity traces. Information on FTIR data analysis including Figure S2 with frequency ν_symm_ as function of the time. Information on alkane viscosity including Figure S3 representing the dynamic viscosity of eicosane and squalane as function of temperature.
