# Supplementary material for: Alkanes as Membrane Regulators of the Response of Early Membranes to Extreme Temperatures
Source: Life (Basel). 2022 Mar 17;12(3):445. doi: 10.3390/life12030445 (PMC8949167; doi:10.3390/life12030445)
Supplement: Supplementary file 1 [file life-12-00445-s001.zip › life-1605428-supplementary.pdf]

life

Article

Supplementary Information

Alkanes as Membrane Regulators of the Response of Early Membranes to Extreme Temperatures

Loreto Misuraca [1,2], Antonino Caliò [3], Josephine G. LoRicco [3], Ingo Hoffmann [2], Roland Winter [4], Bruno Demé [2], Judith Peters [1,2,5,*], and Philippe M. Oger [3,*]

[1] University Grenoble Alpes, CNRS, LIPhy, 38000 Grenoble, France; misuraca.loreto@gmail.com
[2] Institut Laue Langevin, F-38042 Grenoble CEDEX 9, France; hoffmann@ill.fr (I.H.); deme@ill.fr (B.D.)
[3] INSA Lyon, Université de Lyon, CNRS, UMR5240, Villeurbanne, France; antonino.calio@insa-lyon.fr (A.C.); josephineloricco@gmail.com (J.L.);
[4] Fakultät für Chemie und Chemische Biologie, Physikalische Chemie, Technische Universität Dortmund, 44227 Dortmund, Germany; roland.winter@tu-dortmund.de
[5] Institut Universitaire de France, France
* Correspondence: jpeters@ill.fr (J.P.); philippe.oger@insa-lyon.fr (P.O.)

1. SANS data analysis

Form factor models for SANS data analysis:

The following models (and combinations of them, when needed) were used to fit the SANS data:

1.1. Unilamellar vesicle [1]

$$P(q) = scale\,\frac{\Phi}{V_{shell}}\left[\frac{3V_{core}(\rho_{solvent}-\rho_{shell})j_1(qR_{core})}{qR_{core}} + \frac{3V_{tot}(\rho_{shell}-\rho_{solvent})j_1(qR_{tot})}{qR_{tot}}\right]^2 + C \qquad (1)$$

with Φ the shell volume fraction, V the volumes of the core of shell (V_{core}) or the overall vesicle (V_{tot}), R the radii of the core (R_{core}) or the entire vesicle (R_{tot}), ϱ the neutron scattering length densities of solvent ($\rho_{solvent}$) and shell (ρ_{shell}), $j_1(x) = (sinx - cosx)/x^2$ the spherical Bessel function and C the flat background level.

The fitting parameters are therefore the following:

- Φ: determined from the sample preparation and always fixed;
- scale: as the curves are in absolute scale, if all samples having volume fraction Φ contribute to the vesicle form factor, then scale = 1;
- background;
- $\rho_{solvent}$: calculated from $\rho = \frac{\sum_{i=1}^{N} b_i}{V}$ and using tabulated values for the neutron coherent scattering lengths b_i of the atoms in the solvent (buffer and D_2O), always fixed for the fittings;
- ρ_{shell}: same as for $\rho_{solvent}$, calculated from the sample molecules and always fixed;
- R_{core};
- bilayer thickness = $R_{tot} - R_{core}$;

- radius polydispersity, assuming a Lognormal distribution;
- thickness polydispersity, assuming a Gaussian distribution.

1.2. Multilayer vesicle (used for bilamellar vesicle populations)

$$P(q) = scale\,\frac{\Phi(\rho_{shell}-\rho_{solvent})}{V_{shell}}\sum_{i=1}^{N}\left[\frac{3V(r_i)\sin(qr_i)-qr_i\cos(qr_i)}{(qr_i)^3}-\frac{3V(R_i)\sin(qR_i)-qR_i\cos(qR_i)}{(qR_i)^3}\right]^2+C \quad (2)$$

with r_i the solvent radius before shell I, R_i the shell radius for shell i. The fitting parameters are analogous as in the unilamellar vesicle model, except that it is normalised for the vesicle volume fraction (core + shell) and for the additional parameters:
- solvent thickness = r_i - R_{i-1};
- N: number of shells, fixed to 2 for the bilamellar vesicles.

1.3. Lamellar [2] (this model was used when the information about the vesicle size could not be obtained)

$$P(q) = 4\pi\frac{scale}{q^4\delta}\Delta\rho^2(1-\cos q\delta)+C \quad (3)$$

where $\Delta\rho^2$ is the scattering length density difference, and δ the layer thickness. This model can be thought of as the limiting form of a *unilamellar vesicle* model with infinitely large radius.

1.4. Sphere [1] (lipid droplet population)

$$P(q) = \frac{scale}{V}\left[\frac{3V(\rho_{solvent}-\rho_{sphere})(sin(qr)-qrcos(qr))}{(qr)^3}\right]^2+C \quad (4)$$

used to model spheres with uniform neutron scattering length density ρ_{sphere}, radius r and volume V. Free fitting parameters:
- scale: sphere (droplet) volume fraction;
- r: sphere radius.
- radius polydispersity, assuming a Lognormal distribution;

Table S1 (at the end of this text) shows the parameters that have been used for the SANS fit. In every case, the following additional parameters were considered as constant and fixed:

1. Sample neutron scattering length density (NSLD): -0.04 × 10^{-6} Å^{-2}
2. Solvent NSLD: 6.24 × 10^{-6} Å^{-2}
3. Sample volume fraction: 0.0127

The NSLD values were calculated from the compound chemical formula and the density using SASView (http://www.sasview.org/, version 4.2.2, accessed on 17 March 2022).

2. Neutron Spin Echo: *in-situ* DLS

Figure S1 shows the intensity traces acquired during the *in-situ* DLS measurements on IN15. The data give additional evidence about fusion phenomena occurring at high temperature that cause a significant decrease in the amount of scatterers in solution (to which the intensity is partially linked). We made sure that the intensity of the C10 mix sample, although reduced at T = 70 °C, was still significantly higher than the dark counts.

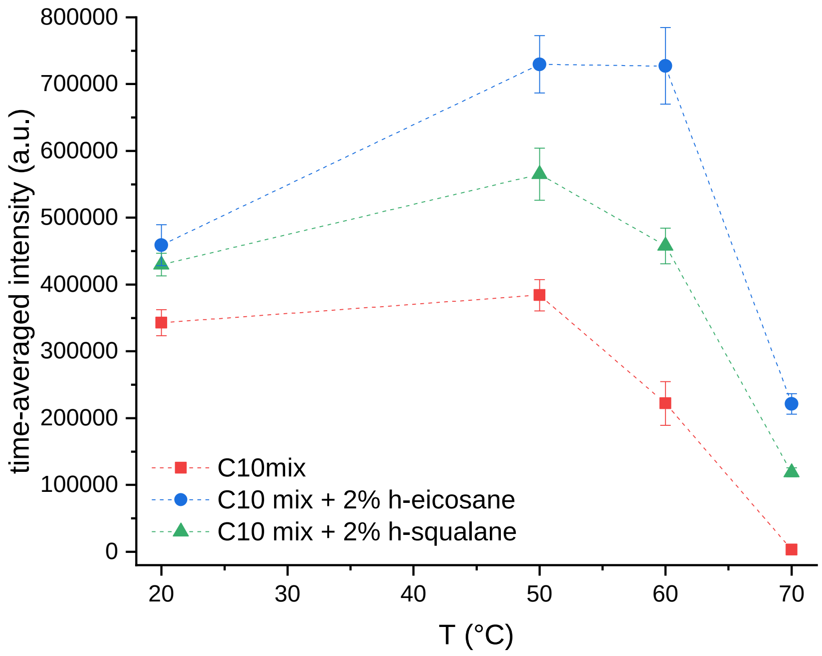

Figure S1. *In-situ* DLS Intensity traces averaged over the full measuring time at each specific temperature for the three measured samples.

3. FTIR data analysis

Figure S2 shows the same data presented in Figure 6 (right) in the main text, but on a wider ν range to show the peak coexistence (and the position of the second peak at $\nu_{symm} \approx 2861$ cm^{-1}) observed at T = 20 °C at the beginning and the end of the scan.

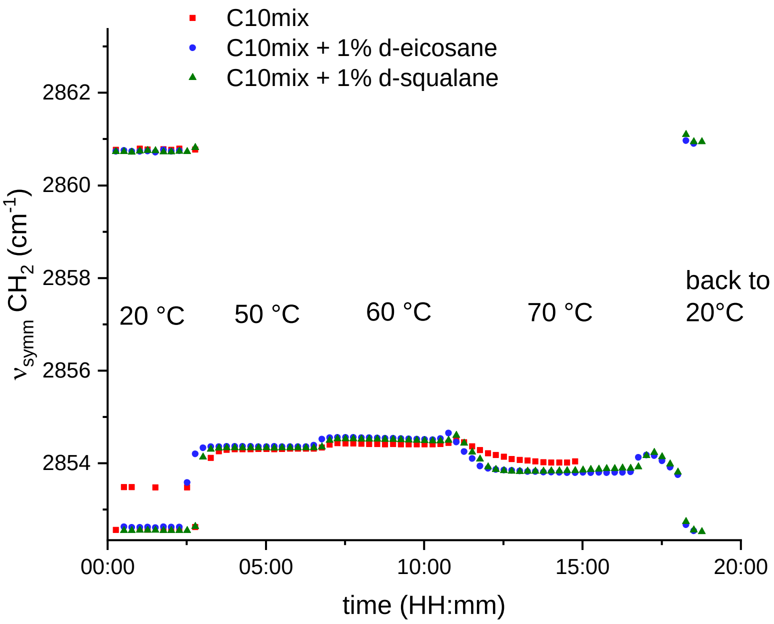

Figure S2. Frequency ν_{symm} as function of the time, following various temperature-jumps for all the samples (similar to Figure 6 in the main text, but here showing the position of the coexisting peak at $\nu_{symm} \approx 2861$ cm^{-1}).

4. Alkane viscosity

Figure S3 plots values of the dynamic viscosity of eicosane and squalane as function of temperature, taken from the literature [3,4]. The difference in the vesicle behavior in the low and the high temperature ranges may be linked to the fluidity of the incorporated alkane molecules.

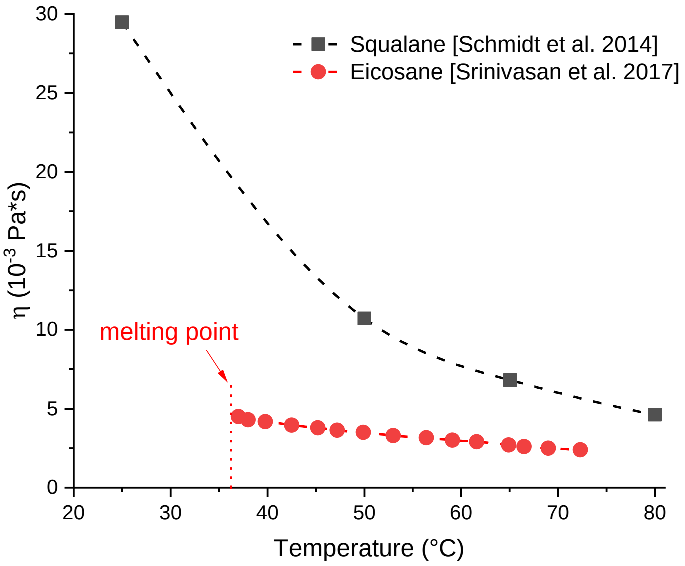

Figure S3. Dynamic viscosity of eicosane and squalane as function of temperature. Data taken from [3,4].

Table S1. Full list of SANS fit parameters. The values for the fractions (lamellar and sphere) are normalized to the lipid volume fraction.

Sample	C10 mix				C10 mix + 2% h-eicosane				C10 mix + 2% h-squalane			
**Temperature (°C)**	**20.5**	**39.7**	**60.1**	**78.7**	**20.5**	**39.7**	**60.1**	**78.7**	20.5	39.7	60.1	78.7
Fitting model	Lamellar (ULV + BLV)	Lamellar (ULV + BLV)	Sphere + lamellar	sphere	Lamellar (ULV + BLV)	Sphere + lamellar (ULV)	lamellar	sphere	Sphere + lamellar (ULV)	Sphere + lamellar (ULV)	lamellar	sphere
q-range ($Å^{-1}$)	all	all	all	all	all	all	0.04 – 0.57	all	all	all	0.04 – 0.57	all
Chi^2/(Npts)	6.1	4.9	3.6	2	23	46.4	4.8	83.1	17.9	22.1	2.8	8.6
Lamellar fraction*10^{-3}	239.0 ± 0.8	245.2 ± 0.9	7.4 ± 0.2	--	869 ± 1	176.8 ± 0.2	186.0 ± 0.6	--	478.3 ± 0.6	358.9 ± 0.7	50 ± 2	--
Vesicle radius (Å)	412 ± 2 (ULV) ; 154 ± 6 (BLV)	433 ± 2 (ULV) ; 164 ± 6 (BLV)	--	--	389 ± 3 (ULV) ; 157 ± 3 (BLV)	119 ± 1	--	--	397 ± 3	175 ± 2	--	--
Membrane thickness (Å)	21.8 ± 0.1	21.8 ± 0.1	20 (fixed)	--	23.6 ± 0.1	21.5 ± 0.1	20.1 ± 0.1	--	22.2 ± 0.1	20.5 ± 0.1	16 ± 1	--
Solvent thickness (for BLV) (Å)	115.8 ± 0.5	130.3 ± 0.6	--	--	69.8 ± 0.6	--	--	--	--	--	--	--
Sphere fraction*10^{-3}	--	--	85.0 ± 0.2	58.8 ± 0.2	--	154.3 ± 0.2	--	424.8 ± 0.3	458.3 ± 0.2	156.1 ± 0.2	--	278.1 ± 0.2
sphere radius (Å)	--	--	768 *	430 *	--	281 *	--	420*	516 *	439 *	--	425 *
Vesicle radius PD	0.19 (ULV) ; 0.54 (BLV)	0.18 (ULV) ; 0.48 (BLV)	--	--	0.30 (ULV) ; 0.67 (BLV)	0.62	--	--	0.45	0.59	--	--
Sphere radius PD	--	--	1	1	--	1	--	1	0.13	0.70	--	1
Solvent thickness PD (for BLV)	0.22	0.24	--	--	1	--	--	--	--	--	--	--

ULV: Unilamellar Vesicle; BLV: Bilamellar Vesicle; PD: polidispersity index. * Since the Guinier regime is not fully reached at low q for the dense sphere form factors, the radius values found from the fits are not reliable and should be considered as minimum values instead.

Reference

1. Guinier, A.; Fournet, G. *Small-Angle Scattering of X-Rays*; John Wiley and Sons Chapman & Hall, Ltd.: New York, London, 1955.
2. Nallet, F.; Laversanne, R.; Roux, D. Modelling X-ray or neutron scattering spectra of lyotropic lamellar phases : interplay between form and structure factors. *J. Phys. II France.* **1993**, *3*, 487–502.

3. Schmidt, K.A.G.; Pagnutti, D.; Curran, M.D.; Singh, A.; Trusler, J.P.M.; Maitland, G.C.; McBride-Wright, M. New Experimental Data and Reference Models for the Viscosity and Density of Squalane. *J. Chem. Eng. Data* **2015**, *60*, 137–150.
4. Srinivasan, S.; Diallo, M.S.; Saha, S.K.; Abass, O.A.; Sharma, A.; Balasubramanian, G. Effect of temperature and graphite particle fillers on thermal conductivity and viscosity of phase change material n-eicosane. *Int. J. Heat Mass Transf.* **2017**, *114*, 318–323.
